# Emotion Recognition from Multiband EEG Signals Using CapsNet

**DOI:** 10.3390/s19092212

**Published:** 2019-05-13

**Authors:** Hao Chao, Liang Dong, Yongli Liu, Baoyun Lu

**Affiliations:** School of Computer Science and Technology, Henan Polytechnic University, Jiaozuo 454000, China; 211709020013@home.hpu.edu.cn (L.D.); liuyongli@hpu.edu.cn (Y.L.); luby@hpu.edu.cn (B.L.)

**Keywords:** EEG signal, feature extraction, multiband feature matrix, deep learning, CapsNet, emotion recognition

## Abstract

Emotion recognition based on multi-channel electroencephalograph (EEG) signals is becoming increasingly attractive. However, the conventional methods ignore the spatial characteristics of EEG signals, which also contain salient information related to emotion states. In this paper, a deep learning framework based on a multiband feature matrix (MFM) and a capsule network (CapsNet) is proposed. In the framework, the frequency domain, spatial characteristics, and frequency band characteristics of the multi-channel EEG signals are combined to construct the MFM. Then, the CapsNet model is introduced to recognize emotion states according to the input MFM. Experiments conducted on the dataset for emotion analysis using EEG, physiological, and video signals (DEAP) indicate that the proposed method outperforms most of the common models. The experimental results demonstrate that the three characteristics contained in the MFM were complementary and the capsule network was more suitable for mining and utilizing the three correlation characteristics.

## 1. Introduction

Emotion is an affective state of human beings accompanied by cognition and consciousness, and plays an important role in human social interactions. Human emotion recognition has become an important research focus in many fields, such as cognitive science, computer science, psychology, neuroscience, and artificial intelligence.

Emotion states can be inferred by the external and internal reactions of a human being, which change with different emotional states. Current emotion recognition research is implemented mainly according to non-physiological signals such as facial expressions [1], speech [2], body movement [3], and physiological signals like skin resistance (SR), electrocardiogram (ECG), functional magnetic resonance imaging (fMRI), electroencephalograph (EEG) [4], and magnetoencephalogram (MEG) [5]. Compared with non-physiological signals, physiological signals are not susceptible to the impact of external environment and subjective will. Therefore, physiological signals—especially EEG signals—become attractive because of the repeatability and objectivity for the estimation of emotion state. In recent years, with the development of sensor technology, it has become possible to monitor, record, and analyze multi-channel neurophysiological signals synchronously. Thus, many researchers have turned their attention to multi-channel EEG-based emotion recognition [6,7,8,9,10,11,12].

Various EEG features have been proposed for emotion recognition in previous studies. Time-domain analysis is implemented to obtain the characteristics of time series which describe salient information related to emotional states. The statistical features (power, mean, standard deviation, etc.) of EEG series are usually employed [13,14,15]. Frantzidis et al. [16] used the event-related potential (ERP) features. In addition, other studies introduced Hjorth features (Mobility, Complexity, and Activity) [17], non-stationary index (NSI) [18] and higher-order crossing features (HOCs) [19,20] for EEG emotion recognition. Power-related measures from different frequency bands of EEG signals are often used in frequency-domain techniques [21]. The most commonly used analytical technique for EEG signals is the fast Fourier transform (FFT) [22,23]. Since the FFT cannot reflect temporal information in the frequency data, the short-time Fourier transform (STFT) is used to extract time-frequency domain features [8,24,25]. Welch’s method [26] was also used to calculate the power spectral density (PSD) of different frequency bands in some studies [27,28].

The choice of specific EEG channels is critical for multi-channel EEG-based emotion recognition. Wichakam et al. [28] made use of all 32 channels as well as 10 specific channels (F3, F4, Fp1, Fp2, P3, P4, T7, T8, O1, and O2) for emotion recognition. The experimental results showed that better results were achieved when using the 10 channels compared to using all 32 channels. Jie et al. [29] proposed an emotion recognition method based on sample entropy. Their experimental results showed that the channels related to emotion states were mainly located in the prefrontal region, namely, F3, CP5, FP2, FZ, and FC2.

Many machine-learning techniques have been applied to identify emotion states, such as support vector machine (SVM) [28], k-nearest neighbor (k-NN) [30], and artificial neural network (ANN) [31]. In recent years, deep learning methods have been introduced for EEG emotion recognition [32,33,34]. Compared with traditional machine learning methods, deep learning methods extract features automatically based on big data and are more capable of portraying the rich intrinsic information of data. Many studies have shown the superiority of deep learning methods in emotion recognition. Kwon et al. [23] proposed a method in which time-frequency feature maps of each channel as well as other physiological features were fed into a convolutional neural network (CNN) to recognize emotion states. Li et al. [7] used frequency features to construct EEG multi-dimensional feature images. Then, a hybrid deep learning model which integrated CNN and recurrent neural network (RNN) techniques was designed to deal with the multi-dimensional feature images in the emotion recognition task.

Compared with traditional machine learning methods, deep learning has demonstrated its potential in multi-channel EEG-based emotion recognition. However, two challenges remain. Firstly, most of the attention is given to determining how to obtain the salient information related to emotional states from the time-domain, frequency-domain, and time-frequency characteristics of EEG signals. Few studies have analyzed the spatial domain characteristics of multi-channel EEG, which may also contain salient information. Further, there have been a few spatial characteristics studies limited to the asymmetry between the electrode pairs [26]. Therefore, determining how to integrate and present the common characteristics of the EEG signal with the spatial characteristics is a key problem. In addition, many deep learning methods have poor sensitivity to spatial domain characteristics in processing two-dimensional (2D) objects, such as the deep belief network (DBN) and the stacked autoencoder (SAE). Although CNNs can handle two-dimensional objects, they lack the ability to describe the relative relationship between the local parts and the whole object, which can provide useful information for classification purposes.

To address the challenges mentioned above, this paper proposes an EEG emotion recognition framework that integrates a multiband feature matrix and a capsule network (CapsNet). This is the first time that CapsNet has been employed for emotion recognition. In the framework, the PSD features are extracted from the EEG signals and multiband feature matrices (MFMs) are constructed according to the electrode positions and frequency bands. Then, MFMs are fed into a CapsNet to perform classification tasks.

The remainder of this paper is organized as follows. Section 2 describes the dataset and emotion model. In Section 3, the extraction method of MFMs and the CapsNet-based framework are introduced. Section 4 analyzes the experimental results and provides discussions. Section 5 summarizes this work.

## 2. Materials

### 2.1. EEG Dataset

The DEAP dataset was employed to validate the effectiveness of the proposed emotion recognition framework [35]. The DEAP dataset recorded several physiological signals and video signals from 32 volunteers when they were watching 40 music videos. Forty trials were implemented for each volunteer. Each trial played a specific 63-s video, with the EEG and peripheral physiological signals simultaneously recorded. After watching the videos, the subjective ratings of arousal, valence, liking, and dominance on a scale of 1–9 were gathered. EEG data of 512 Hz sampling frequency were recorded with 32 electrodes placed according to the international 10/20 system. Each electrode recorded a 63-s EEG signal, and the first 3 s was the baseline signal of the trail.

In this paper, the preprocessed raw EEG data and the corresponding emotion self-assessment in the DEAP dataset were used. EEG signals (512 Hz) were down-sampled to 128 Hz. The effects of the electrooculogram (EOG) were removed and band-pass filtering was implemented with cutoff frequencies of 4.0 and 45.0 Hz.

### 2.2. Emotion Model

**Figure 1:**
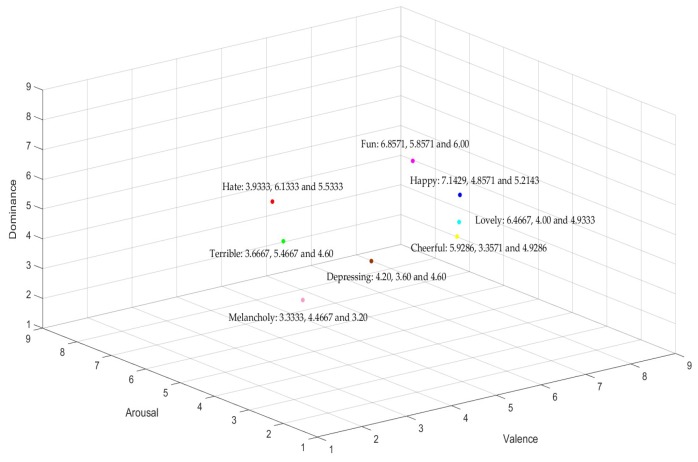
Three-dimensional space of the emotion model.

This work relies on the valence–arousal–dominance space model because of its simplicity and ability to measure emotions relatively well [36]. Figure 1 shows the three-dimensional emotion model with valence, arousal, and dominance. The model explains emotions’ change in a 3D plane, and the emotion states are determined by the values of arousal, valence, and dominance. As shown in Figure 1, the values of an emotional instance of Fun in valence, arousal, and dominance dimensions are 6.8571, 5.8571, and 6.00 respectively.

**Figure 2:**
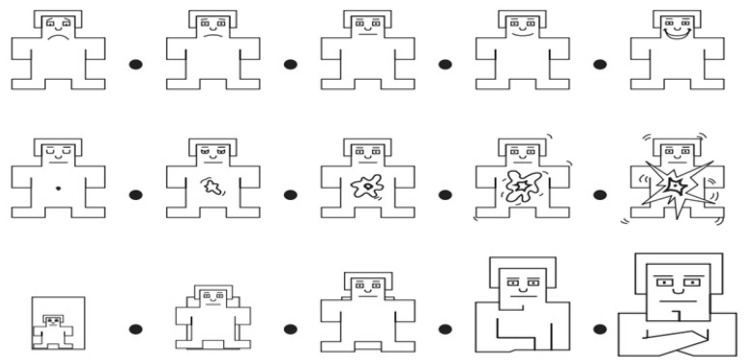
Images used for self-assessment: (**top**) Valence self-assessment manikin (SAM); (**middle**) Arousal SAM; (**bottom**) Dominance SAM.

In the DEAP dataset, at the end of each trial, participants performed a self-assessment of their levels of arousal, valence, and dominance. Self-assessment manikins (SAMs) [37] were used to visualize the scales (see Figure 2). The manikins were displayed with the numbers 1–9 printed below. Participants were informed that they could click anywhere directly below or in-between the numbers, making the self-assessment a continuous scale [35]. In other words, the ratings given by each participant were real numbers within the interval 1–9. Therefore, thresholds need to be used to produce class labels. More categories can describe emotions more accurately. However, it greatly increases the difficulty of emotion recognition. In the preliminary stage of affective computing, many emotion recognition studies used label-processing methods based on self-assessment ratings of 1–5 and 5–9 [9,38,39,40,41,42]. Koelstra et al. [35] divided the task of recognizing emotion into different binary classification problems. Wang et al. [42] carried out a binary classification task in the two dimensions of arousal and valence.

There are three indicators: arousal, valence and dominance. Each trial was divided into two classes on each indicator. If the personal rating was less than 5, the label was set to “low”. If it was greater than or equal to 5, the label was set to “high” [9,38,39,40,41,42]. Thus, there were a total of six labels: HA (high arousal), LA (low arousal), HV (high valence), LV (low valence), HD (high dominance), and LD (low dominance) in three dimensions. Specifically, we divided the emotion recognition task into three binary classification problems.

## 3. Methods

### 3.1. Multiband Feature Matrix

**Figure 3:**
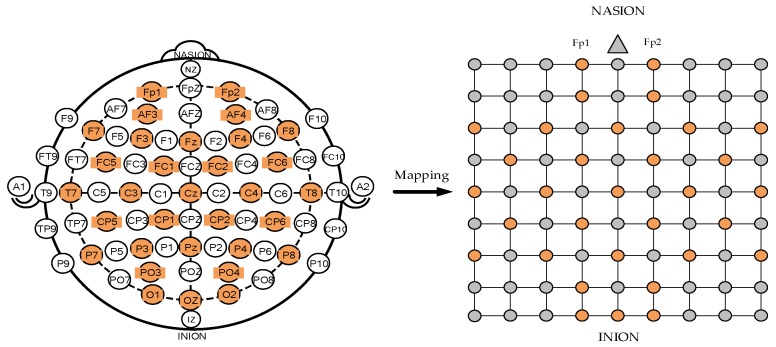
International 10–20 system and 9 × 9 square matrix.

The international 10–20 system describes the position of the scalp electrodes, and it is based on the relationship between electrode positions and the underlying region of the cerebral cortex. The “10” and “20” refer to the fact that the actual distances between adjacent electrodes are either 10% or 20% of the total front–back or the right–left distance of the skull. Figure 3 shows the specific form of the International 10–20 System and its mapping on a square matrix. The electrode positions marked with orange on the left side are the electrodes contained in the DEAP dataset. In this study, the 32 electrodes used in the DEAP dataset were mapped into a 9 × 9 square matrix. The orange points in the grid matrix represent the electrodes used in the international 10–20 system, and are filled with the frequency feature (PSD) of the EEG signals. The gray points are unused electrodes and are filled with default values. The nasion is represented by a gray triangle above the square matrix.

**Figure 4:**
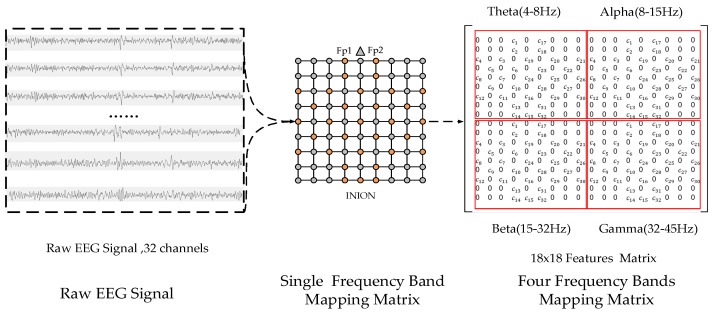
The mapping process of multiband feature matrix according to the raw electroencephalograph (EEG) signals of 32 channels.

Fusion of the power features from different frequency bands has been used in emotion recognition studies [4,6]. Therefore, multiple sub-matrices were constructed to describe the salient information related to the emotion states in different frequency bands, and each sub-matrix corresponded to a frequency band. Then, these sub-matrices were merged according to specific rules to structure the MFM. The MFM construction process is presented in Figure 4.

The power spectral density (PSD) was extracted from the raw signal as a frequency domain feature. The PSD of each channel was divided into four parts according to the four frequency bands of theta (4–8 Hz), alpha (8–15 Hz), beta (15–32 Hz), and gamma (32–45 Hz). The average PSD value of each frequency band was taken as a frequency-domain feature value. Thus, four values were obtained for one channel. The number of features for each sample was 128 (32 channels and |4 frequency bands). The features of each subject were normalized using Equation (1), and the interval was from 0 to 1:

(1) $$F^{\prime }=\frac{F_{\max}-F}{F_{\max}-F_{\min}},$$

where $F^{\prime }$ is the normalized feature value. $F_{\max}$ and $F_{\min}$ represent the maximum and minimum feature values, respectively. *F* is the feature value before normalization.

After normalization, each sample took the features of 32 channels with the same frequency band and constructed a sub-matrix according to the mapping rules described in Figure 3. The element values in the sub-matrix were set to the average PSD of the corresponding channels, and elements without corresponding electrodes in the sub-matrix had a default value of 0.

Considering that there are four bands (i.e., theta, alpha, beta, and gamma), four sub-matrices were obtained for each sample. The four sub-matrices were combined into an MFM in a 2 × 2 manner, as shown in Figure 4. Thus, an MFM of size 18 × 18 was constructed. In each sub-matrix, $c_{j}$ represents the PSD value of the *j*-th channel in the corresponding frequency band.

In the MFM, the elements in each sub-matrix used to represent the EEG channels were consistent with the positions of sensors placed on the scalp when the EEG signals were acquired. The elements’ values were the average PSD values of the corresponding channels. The MFMs showed the variations of human EEG signals on the scalp directly and accurately. Compared with traditional features, MFMs—which combine the frequency domain, spatial, and frequency band characteristics of multi-channel EEG signals—can provide richer information representing EEG signal variations in different emotion states.

### 3.2. Capsules Network

**Figure 5:**
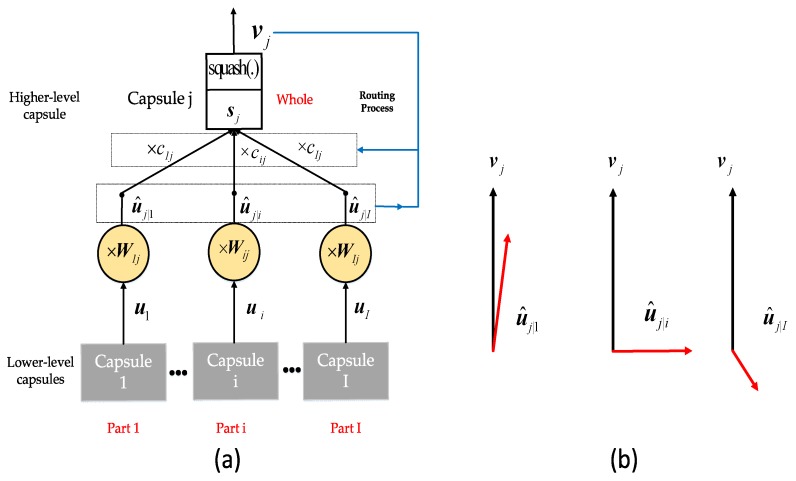
(**a**) Information transfer between capsules; (**b**) Routing process.

The CNN is the most commonly used method for two-dimensional object classification. However, information such as position and pose in the object is discarded by the CNN due to its data routing procedure. To compensate for the shortcomings of CNN, a network structure called the capsule network has been proposed [43]. CapsNet is a deep network model consisting of capsules. A capsule is composed of a group of neurons. Activation neurons represent the characteristics of components in the object. Each capsule is responsible for determining a single component in the object, and all capsules jointly determine the overall structure of the object. In contrast to some deep neural networks (e.g., DBN), this structure preserves objects’ components and spatial information. Similar to CNN, a CapsNet is composed of a multi-layer network. Figure 5 shows the structure of a capsule and how information is routed from the lower-level capsules to the subsequent higher-level capsules.

In part (a) of Figure 5, the inputs and outputs of the capsules are vectors. The length of the output $u_{i}$ represents the probability of existence of its corresponding component, and the directions of the vector $u_{i}$ encode various properties (e.g., size, position) of its corresponding component. The predictive vector ${\hat{u}}_{j|i}$ represents the belief, which encodes the relationship between the *i*-th capsule in the lower-level capsules and *j*-th capsule in the higher-level capsules using a linear transformation matrix $W_{ij}$ in Equation (2):

(2) $${\hat{u}}_{j|i}=W_{ij}\cdot u_{i}.$$

That is, the detected component’s existence and pose information are used to predict the whole presence and pose information. During the training process, the network gradually learns to adjust the transformation matrix of the capsule pair through the corresponding relationship between components and the whole in the object.

In the higher-level capsules, $s_{j}$ and $v_{j}$ are the input and output of capsule *j*, respectively. $s_{j}$ represents the sum values of the predicted vectors ${\hat{u}}_{j|i}$ with corresponding weight $c_{ij}$ in low-level capsule *i*. In Equation (3), $c_{ij}$ is the coupling coefficient and is determined by an iterative dynamic routing algorithm, where $\sum_{j}c_{ij}=1$ and $c_{ij}\geq 0$. When $c_{ij}=0$, there is no information transfer between capsule *i* and capsule *j*, whereas when $c_{ij}=1$, all the information of capsule *i* is transmitted to the high-level capsule *j*. Since the length of the output represents a probability value, a non-linear squash function is used to ensure that the short vector is compressed to be closer to 0 and the long vector is compressed to be closer to 1. The squash function is shown in Equation (4):

(3) $$s_{j}=\sum_{i}c_{ij}\cdot {\hat{u}}_{j|i},$$

(4) $$v_{j}=\frac{\left\|s_{j}^{2}\right\|}{1+\left\|s_{j}^{2}\right\|}\frac{s_{j}}{\left\|s_{j}\right\|},$$

(5) $$c_{ij}=\frac{\exp(b_{ij})}{\sum_{k}\exp(b_{ik})},b_{ij}\leftarrow b_{ij}+{\hat{u}}_{j|i}\cdot v_{j}.$$

As shown in part (b) of Figure 5, when the lower-level capsules and the higher-level capsules are consistent with their predictions, the value of $c_{ij}$ becomes larger by Equation (5), and it gets smaller when they are inconsistent. By adjusting the routing coefficient, the dynamic routing algorithm ensures that the lower-level capsules send their prediction vectors to the higher-level capsules that are consistent with their predictions, so that the outputs of the sub-capsules are sent to the correct parent capsules. The specific process of the dynamic routing algorithm can be found in [43].

### 3.3. Capsule-Network-Based Deep Learning Model

A CapsNet can encode the relative relationship between local parts and whole objects (e.g., scale, position, direction), which is discarded by CNNs in the process of max-pooling and average-pooling. Therefore, CapsNet has a natural understanding of three-dimensional space and contains the states and relative positional relationships between all components of an object. Thus, it is more sensitive to differences in spatial information between different objects. The components in an image are represented by the values of the pixels in the corresponding region. Similarly, in different emotional states, different regions of the cerebral cortex have corresponding responses. Corresponding to the MFM, this is the features of EEG channels in different regions. In view of this, CapsNet can distinguish the salient global difference information reflected by the human brain in different emotional states when it is applied to multi-channel EEG-based emotion recognition. In addition, CapsNet has a faster learning speed and less sample usage than CNN. Therefore, CapsNet is introduced into EEG-based multi-channel emotion recognition, and a deep learning framework based on an MFM and a capsule network is proposed.

The CapsNet-based model consists of four parts. The first part is convolution with rectified linear unit (ReLU), and convolution operations are performed on the input MFM to detect local features in this part. Primary capsules (PrimaryCaps) is the second part, which consists of a convolution process and transmits information to capsules. The third part is Emotion capsules (EmotionCaps), which includes the dynamic routing process between capsules and is used for emotion recognition. The last part attempts to reconstruct the input matrix from the output of the final capsules.

**Figure 6:**
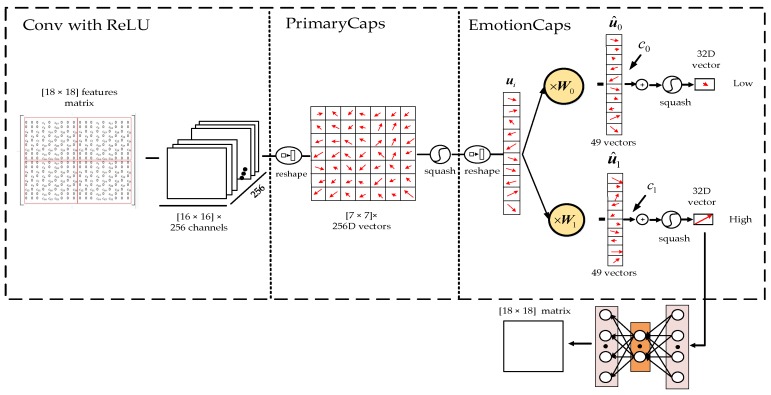
Architecture of the capsule network (CapsNet)-based model. ReLU: rectified linear unit.

Figure 6 describes the structure of the proposed CapsNet-based model. In the convolution with ReLU, an 18 × 18 MFM which is described in Section 3.1 is decoded as the input in the first layer. The first step is the standard convolution operation; a 3 × 3 convolution kernel with a stride size of 1 and a ReLU activation function is employed. This convolutional layer does not use padding, and the output volume is 16 × 16 with 256 channels.

In the PrimaryCaps part, convolution operations are first performed on the data from the previous part, and the convolution layers are composed of a convolution unit with a convolution kernel size of 3 × 3 and a stride of 2. After the convolution operation, the output data are reshaped to 256-dimensional (256D) vectors in 7 × 7 arrays. Then, the squash activation function is applied.

The EmotionCaps part reshapes the 7 × 7 arrays generated from the PrimaryCaps, and forms 49 by the 256D vector. Then, the vector is multiplied by weight matrix $W_{i}$, and the vector ${\hat{u}}_{i}$ can be obtained, where *i* denotes the index of each output class. Then, $c_{i}$ is determined by the dynamic routing algorithm. The dynamic routing algorithm iterates three times in this model, and $c_{i}$ encodes the ${\hat{u}}_{i}$ into a 32-dimensional activation vector of instantiation parameters. Lastly, the output vector is squashed in order to determine the probability of each emotion state. The values of *i* are 0 and 1, which correspond to the respective emotion classes.

The final part attempts to reconstruct the input MFM from the final capsules, which keeps the information from the input as much as possible throughout the network. Thus, it can prevent overfitting and help to generalize new samples as a normalizer. The structure uses a three-layer feed-forward neural network with 512, 1024, and 324 units, respectively. This model uses L2 for reconstruction and margin loss for classification. The margin loss is shown in Equation (6):

(6) $$L_{e}=T_{e}\max{\left(0,m^{+}-∥v_{e}∥\right)}^{2}+\lambda (1-T_{e})\max{\left(0,∥v_{e}∥-m^{-}\right)}^{2},$$

where $T_{e}$ equals 1 if an emotional class *e* is present and $m^{-}$ = 0.1 and $m^{+}$ = 0.9. The λ is down-weighting of the loss and it was set to 0.5 by default. $v_{e}$ represents the final output vector of class *e*.

## 4. Results and Discussions

In order to increase the number of samples, the raw EEG signals of each channel were divided into 20 sections by using a sliding window. The duration of the sliding window was 3 s, and there was no overlap region between adjacent windows. Each section was regarded as an independent sample, and inherited the labels of the original sample. The number of EEG samples per subject was 800 (40 × 20), and the number of all samples was 25,600 (800 × 32). The total number of MFMs of all subjects was 25,600, corresponding to the number of samples, as shown in Table 1.

### 4.1. Selection of Hyperparameters

Each sample constructed a corresponding MFM. Since MFMs of single subjects are not sufficient for training a stable recognition model, the MFMs of all subjects were used to improve the generalization ability of the proposed method.

The classification performance was analyzed via a 10-fold cross validation technique. For this work, the 25,600 MFMs were divided into 10 subsets. Nine subsets were assigned to the training set, and the remaining one was assigned to the test set. The above process was repeated 10 times until all subsets were tested. The training epochs and batch size for each experiment were set to 400 and 40, respectively. Dynamic routing algorithms in all models were iterated three times.

Different combinations of hyperparameters and training parameters were tested to determine the appropriate parameter combination of the proposed model, and the parameter combination with the minimal recognition error was adopted. Considering that the computational complexity of the proposed model will increase sharply as the number of layers increases, the hyperparameters included only the parameters of the first convolution layer, filters of PrimaryCaps, and parameters of EmotionCaps. The number of layers of the CapsNet remained unchanged.

The number and form of trainable parameters affect the performance of the capsule network. To find the optimal combination of model parameters, a two-pass search process from coarse to fine was adopted. Firstly, we made a coarse selection to determine the scope of the number of model parameters. The network parameters were selected by referring to references [43,44]. Table 2 shows the network models with different numbers of parameters in the coarse selection step.

**Figure 7:**
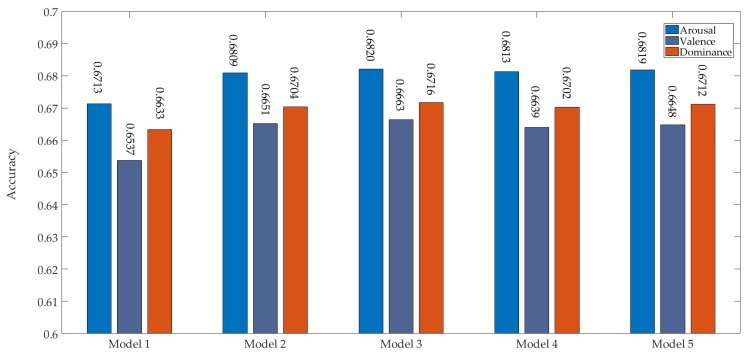
Accuracy of different models in three dimensions in the first step.

Figure 7 shows the emotion recognition results of the models in the coarse selection step. Compared with model 1, model 2 and model 3 achieved better recognition performance in three dimensions. The reason may be that model 1 had fewer parameters, so it could not fully learn the features of MFMs. The four remaining models had similar recognition performance. However, model 3 and model 4 had higher computational complexity. Therefore, model 2 and model 3 were analyzed further.

When the scope of the number of parameters was determined, several models which had similar numbers of parameters and different network structures were employed to determine the optimal parameter combination. Specifically, we adjusted the parameters of the three main parts of model 2 and model 3, including the size of the convolution kernel in the first part, the parameters of the Primary part, and the parameters of the EmotionCaps part. Table 3 shows the combination of parameters used by different network models.

**Figure 8:**
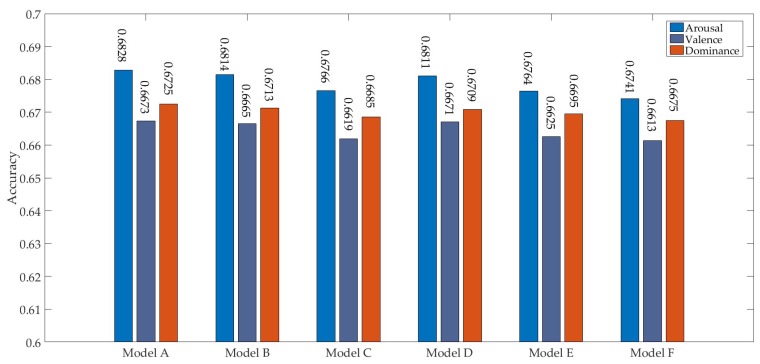
Accuracy of different models in three dimensions in the second step.

Figure 8 shows the emotion recognition results of the evaluation method by using different models in the second step. Model A achieved the highest recognition accuracies in all three dimensions (i.e., 0.6828, 0.6673, and 0.6725 in arousal, valence, and dominance, respectively). The reason may be that the output volumes from the first convolution operation of the model A were larger, which is more beneficial for preserving the features of the matrix. Furthermore, the vectors of model A in EmotionCaps layer had higher dimension, and also contained more spatial information. The training speeds of models A, B, D, and E were more than twice those of models C and F. Thus, the hyperparameters of model A were adopted. It can be seen from the comparison of the models that the output volume of the first convolutional layer and the vectors of the PrimaryCaps had an influence on the classification performance.

### 4.2. Comparison between the CapsNet Model and Common Classifiers

**Figure 9:**
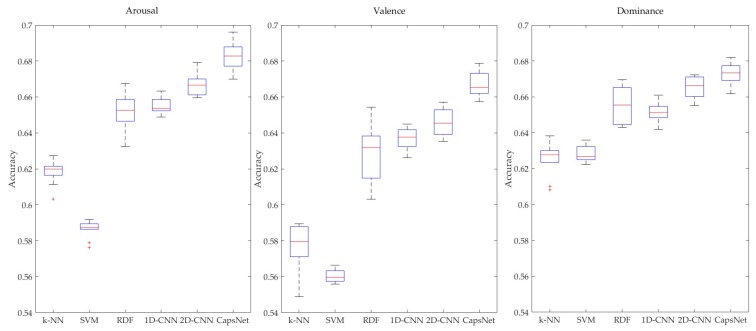
Comparison of emotion recognition results between CapsNet and comparison classifiers. CNN: convolutional neural network; k-NN: k-nearest neighbor; RDF: random decision forest; SVM: support vector machine.

To verify the effectiveness of the CapsNet-based model in utilizing frequency-domain, spatial-domain, and frequency-band characteristics of multi-channel EEG signals for emotion recognition, we also compared the average recognition performance between the CapsNet-based model and several common models which also use MFM as the input feature (Figure 9). These models included 1D-CNN, 2D-CNN, random decision forest (RDF), k-nearest neighbor, and support vector machines. All methods used a 10-fold cross validation technique, and 25,600 MFMs were employed.

Specifically, 1D-CNN and 2D-CNN were implemented by TensorFlow and the structures were similar to the capsule network constructed in the proposed model. In the 1D-CNN model, the input data were 128-dimensional vectors (32 channels, 4 frequency bands) that were not rearranged. The first convolution layer employed a 1 × 9 kernel with a stride size of 1 and a ReLU activation function. The number of filters in this layer was 256. The second convolutional layer had the same parameters as the first convolutional layer. The third layer also had the same convolutional parameters as the first layer, except that the number of filters was 128. Each convolutional layer corresponded to a max pooling layer, which implemented a max pooling process for information aggregation after the convolution operation was completed. The parameters of the three max pooling layers were the same. Each max pooling layer used a 1 × 2 kernel with a stride size of 1 and its padding was “SAME”. The node numbers of the two full-connection layers were 324 and 162, respectively, and the output layer used a softmax function. In the 2D-CNN model, the convolution layer employed a 5 × 5 kernel and the max pooling layer used a 3 × 3 kernel. All parameters except those mentioned above were the same as for the 1D-CNN.

Other common models include RDF, which is based on ensemble learning theory; k-NN, which is based on distance metric (*neighbors* = 5); SVM, based on statistical learning theories (*c* = 10; *kernel* = linear). The above three models were implemented by the Scikit-learn toolkit [45]. In order to adapt to the data entry forms of the above classifiers, each row in the matrix was extracted to form a vector, and all vectors were connected to form a high-dimensional vector.

The recognition results of the CapsNet model A and the five other common models are illustrated in box plots in Figure 9. The average recognition accuracies of all subjects using CapsNet model A in the three dimensions of arousal, valence, and dominance were 0.6828, 0.6673, and 0.6725, respectively, whereas the average accuracies of other models were 0.6667, 0.6460, and 0.6658 with 2D-CNN; 0.6550, 0.6370, and 0.6513 with 1D-CNN; 0.6512, 0.6288, and 0.6547 with RDF; 0.5862, 0.5603, and 0.6286 with SVM; and 0.6182, 0.5770, and 0.6254 with k-NN. The recognition accuracies of 2D-CNN in three dimensions were higher than that of 1D-CNN. It can be seen that the rearranged features (MFMs) could provide additional spatial information representing EEG signal variations in different emotion states. The CapsNet model had the highest average accuracy. In addition, the difference between all experimental results of this method was relatively smaller than that of other methods. The reason may be that the proposed model based on a capsule network can make better use of the three features contained in the MFM and has stronger generalization ability. Compared with CapsNet, 1D-CNN and 2D-CNN could not retain salient spatial information between all channels and bands. Therefore, the classification accuracies of the two CNN models were lower. Due to the architecture and fundamentals of RDF, k-NN, and SVM, the MFM is converted into a high-dimensional vector. Therefore, they can only distinguish the difference of frequency-domain features in the same channels and cannot mine the salient spatial information between channels in different frequency bands, so their recognition performance was poor. We also applied a Wilcoxon signed-rank test (∂<0.05) to examine the performance between CapsNet and 2D-CNN in three dimensions. The null hypothesis was that the performance was similar. If the *p*-value was larger than ∂, the null hypothesis was accepted. The *p*-values of arousal, valence, and dominance were 0.002, 0.002, and 0.0488, respectively. The results of the Wilcoxon signed-rank test showed that the performance of CapsNet was superior to 2D-CNN, with *p*-values less than 0.05 in three dimensions. The results show that spatial differences between channels in different frequency bands could provide salient information regarding emotion states and the model based on capsules network proposed in this paper could utilize the above information effectively.

**Figure 10:**
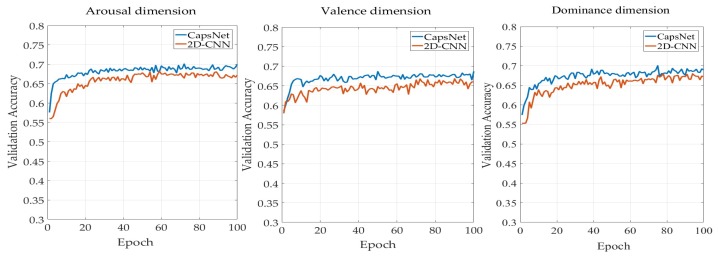
Comparison of 2D-CNN and CapsNet using multiband feature matrices (MFMs).

In view of the similarity between 2D-CNN and CapsNet, hold-out validation was used to evaluate the two models for further comparison. The 25,600 MFMs were randomly divided into training, validation, and test sets, with respective proportions of 8:1:1 for each label. The results in the validation set are shown in Figure 10. With the same data, the validation accuracy of CapsNet increased faster in three dimensions, and the accuracy of CapsNet was higher than that of 2D-CNN with a similar network structure using the same epoch.

### 4.3. Comparison between the Proposed Methods and Several Existing Studies

Finally, we compared the proposed method with other methods using the same dataset. Table 4 shows the details of the comparison methods. Among them, dual-tree complex wavelet packet transform (DT-CWPT) time-frequency features were used in references [38,39]. Items crossed in the table are not indicated in the corresponding references.

As shown in Table 4, the proposed method achieved the highest recognition accuracy in arousal and valence dimensions, with the exception of the results of reference [42]. It can be seen that the proposed method could improve the performance of multi-channel EEG-based emotion recognition. The recognition accuracy in valence and arousal dimensions of reference [42] was higher than that in this work. The reason may be that the method in reference [42] constructs a subject-related classification model, which was only employed to recognize the samples belonging to the corresponding subject. Although the recognition accuracy of references [38] and [39] in the dominance dimension was higher than that of our work, it was lower in the other two dimensions than our method. In order to improve the generalization of the model, the proposed framework used all the data of all subjects for emotion recognition, without considering the differences between the subjects. The results showed that the proposed framework also achieved good performance in all subjects’ EEG-based emotion recognition task. Regardless, the framework based on MFM and CapsNet achieved satisfactory results for EEG emotion recognition.

## 5. Conclusions

In this paper, a framework using multi-channel EEG signals and a deep learning model was proposed to recognize human emotions. The innovation of this work is in two main aspects. Firstly, a novel feature extraction is proposed. Specifically, the frequency-domain features are extracted from EEG signals, and are mapped to an MFM according to electrode positions and frequency band information. The MFM reflects not only the relative position relationship between EEG channels, but also the difference of frequency bands in the EEG frequency domain features. Thus, it provides salient information related to emotion states in spatial and frequency domains. Meanwhile, we also propose a deep learning model based on a capsule network, which was used to judge emotional state. This is the first attempt to apply a capsule network to multi-channel EEG emotion recognition.

The experimental results show that the proposed method achieved satisfactory results in two indicators. This demonstrates that the three kinds of information contained in the proposed MFM features are helpful to recognize human emotion, and the model based on CapsNet could effectively use this information for emotion recognition. At present, our method has only been tested on DEAP datasets. We will test it on more emotional datasets to verify the method comprehensively in the future.

## Figures and Tables

**Table 1:** Distribution of samples. HA: high arousal; HD: high dominance; HV: high valence; LA: low arousal; LD: low dominance; LV: low valence.

Label	Data Quantity	Label	Data Quantity	Label	Data Quantity
HA	14,740	HV	14,160	HD	15,600
LA	10,860	LV	11,440	LD	10,000
Total	25,600	Total	25,600	Total	25,600

**Table 2:** Five CapsNet-based models with different parameters in the first step.

Input Data	Model	Conv. with ReLU	Primary Capsule	EmotionCaps	CapsNet Output
(18 × 18) × 1	1	3 × 3 × 32	3 × 3× (1 × 32)	(7 × 7) × 32	2 × 16
	2	3 × 3 × 128	3 × 3 × (1 × 128)	(7 × 7) × 128	2 × 16
	3	3 × 3 × 256	3 × 3 × (1 × 256)	(7 × 7) × 256	2 × 16
	4	3 × 3 × 384	3 × 3 × (1 × 384)	(7 × 7) × 384	2 × 16
	5	3 × 3 × 512	3 × 3 × (1 × 512)	(7 × 7) × 512	2 × 16

**Table 3:** Six CapsNet-based models (A, B, C, D, E, and F) with different parameters in the second step.

Input Data	Model	Conv with ReLU	Primary Capsule	EmotionCaps	CapsNet Output
(18 × 18) × 1	A	3 × 3 × 256	3 × 3 × (1 × 256)	(7 × 7) × 256	2 × 32
	B	5 × 5 × 256	3 × 3 × (1 × 256)	(6 × 6) × 256	2 × 16
	C	9 × 9 × 256	3 × 3 × (32 × 8)	(4 × 4 × 32) × 8	2 × 32
	D	3 × 3 × 128	3 × 3 × (1 × 128)	(7 × 7) × 128	2 × 32
	E	5 × 5 × 128	3 × 3 × (1 × 128)	(6 × 6) × 128	2 × 16
	F	9 × 9 × 128	3 × 3 × (16 × 8)	(4 × 4 × 16) × 8	2 × 32

**Table 4:** Details of several reported studies. DT-CWPT: dual-tree complex wavelet packet transform.

Studies	Features	Classifiers	Hyperparameters	Evaluation methods	Accuracy		
					Arousal	Valence	Dominance
Koelstra et al. [35]	Power spectral features	Gaussian naive Bayes	_	leave-one-trail-out validation	0.6200	0.5760	_
Naser and Saha [38]	DT-CWPT	SVM	radial-basis kernel	leave-one-trail-out validation	0.6690	0.6530	0.691
Naser and Saha [39]	DT-CWPT	SVM	radial-basis kernel	leave-one-trail-out validation	0.6620	0.6430	0.689
Chung and Yoon [40]	Power spectral features	Bayes classifier	weighted-log-posterior function	10-fold cross-validation	0.6640	0.6660	_
Li et al. [9]	DBN features	SVM	radial-basis kernel	10-fold cross-validation	0.6420	0.5840	0.658
Wang and Shang [41]	DBN features	Deep belief networks	DBN structure: 512–50–50–2 learning rate: 0.05 epochs: 20 Mini-batch: 100	leave-one-trail-out validation	0.5120	0.6090	_
Zhuang et al. [42]	The first difference of time series, etc.	SVM	radial-basis kernel	leave-one-trail-out validation	0.6910	0.7199	_
The proposed method	Multiband Feature Matrix	CapsNet	hyperparameters of the model A	10-fold cross-validation	0.6828	0.6673	0.6725
